# 5D Flow MRI Reveals Respiration‐Driven Changes in Blood Flow Energetics in Congenital Heart Disease

**DOI:** 10.1002/mrm.70403

**Published:** 2026-04-26

**Authors:** Thara Nallamothu, Elizabeth K. Weiss, Justin Baraboo, Joshua D. Robinson, Cynthia K. Rigsby, Christopher W. Roy, Matthias Stuber, Michael Markl

**Affiliations:** ^1^ Radiology, Feinberg School of Medicine, Northwestern Medicine Chicago Illinois USA; ^2^ Biomedical Engineering, Northwestern University Chicago Illinois USA; ^3^ Department of Cardiology Ann & Robert Lurie Children's Hospital Chicago Illinois USA; ^4^ Department of Medical Imaging Ann & Robert Lurie Children's Hospital Chicago Illinois USA; ^5^ Radiology, University Hospital Lausanne (CHUV) Lausanne Switzerland

**Keywords:** 5D flow CMR, congenital heart disease, Fontan, respiratory‐resolved flow, shunt, single ventricle

## Abstract

**Purpose:**

Hemodynamic monitoring is essential for patients with right‐sided congenital heart disease (CHD). Respiration may have an increased impact on pulmonary flow in these patients that cannot be assessed by standard tools including 4D flow MRI. This study uses 5D flow MRI to assess respiratory‐cycle variations in flow energetics in patients with CHD.

**Methods:**

5D flow was acquired with four respiratory states in 14 Fontan patients (21 ± 8 years, 8 female), 10 intracardiac shunt patients (19 ± 13 years, 8 female), and 9 controls (26 ± 6 years, 1 female). Blood kinetic energy (KE_mean_), viscous energy loss (EL_total_), and EL fraction (EL_total_/KE_mean_) as a measure of flow inefficiency were calculated in inferior and superior caval veins (IVC, SVC), pulmonary arteries (PA), and aorta. Correlations were assessed with clinical markers of altered cardiac flow function.

**Results:**

5D flow was acquired with acceleration factor *R* = 37–93 varying between respiratory states. Fontan and shunt patients demonstrated significant respiratory‐driven changes in pulmonary flow energetics compared to controls. Fontan IVC and PA KE_mean_ were increased during inspiration (+43%, +37%, *p* < 0.001) and decreased during expiration (−40%, −35%, *p* < 0.001), resulting in increased expiratory EL fraction (+34%, +30%, *p* < 0.05). Shunt patients showed a similar effect in IVC KE_mean_ (+28%, −25%, *p* < 0.05). Decreased expiratory IVC KE_mean_ was associated with increased Fontan left–right PA flow differential (ρ = −0.68, *p* < 0.05) and increased shunt Qp/Qs (ρ = −0.70, *p* < 0.05).

**Conclusions:**

The findings of this study show that CHD flow energetics and efficiency are modulated by respiration. Respiratory‐resolved imaging is needed to identify these dynamics and their relationships to overall cardiac function.

## 1 Introduction

Patients with complex congenital heart disease (CHD), such as single ventricle defect (SVD) with Fontan repair or with intracardiac shunts, often require hemodynamic monitoring throughout their lives. The standard of care for these patients includes Doppler echocardiography and cardiovascular MRI including flow imaging with 2D phase‐contrast MRI, as well as invasive hemodynamic measurements. These techniques can capture Fontan and shunt velocities to evaluate the risk of life‐threatening outcomes such as Fontan failure and shunt reversal [1, 2, 3, 4, 5, 6]. However, these modalities cannot access the complex 3D changes in blood flow dynamics in SVD and shunt patients [7, 8, 9, 10]. To acquire more detailed flow information, 4D flow MRI has been used as a non‐invasive method for hemodynamic assessment based on the measurement of the 3D velocity vector field of fluid flow throughout the heart. This allows for quantification of advanced measures of complex 3D hemodynamics, such as flow energetics [11, 12, 13]. Previous studies have shown that blood kinetic energy (KE) and viscous energy loss (EL) are associated with poor clinical outcomes in Fontan patients [14, 15, 16, 17, 18, 19]. The dimensionless energy loss fraction (EL/KE) has been proposed to capture a measure of flow inefficiency and has been shown to be elevated in the Fontan connection [12, 15, 20].

Both Fontan and shunt patients experience modulated fluid pressure in right heart circulation. Fontan patients exhibit elevated central venous pressure and lower pulmonary artery (PA) pressure, and patients with left‐to‐right shunts have increased PA pressure but decreased systemic pressure [21, 22, 23]. These differences suggest that changes in chest pressure caused by respiration may have an increased impact on caval venous and pulmonary flows. There is a well‐documented impact of respiration on flow pulsatility in Fontan patients, where thoracic pressure decreases during inspiration drive increased pulmonary‐venous return [24, 25]. In patients with left‐to‐right cardiac shunts, Qp/Qs has been found to change over respiration, with patient‐dependent increases during inspiration versus expiration [26, 27]. However, 4D flow and other modalities only capture a single respiratory state (e.g., end‐expiration) and are thus inadequate to assess the impact of respiration on blood flow dynamics [7, 8, 28]. As a result, 4D flow cannot distinguish changes in hemodynamics due to respiratory‐driven changes in intrathoracic pressure.

5D flow MRI is an emerging technique that can resolve 3D hemodynamics across both the cardiac and respiratory cycles [29]. This technique is free‐running, using golden angle radial sampling and an inherent self‐gating signal to allow for highly accelerated retrospective compressed sensing (CS) reconstruction of flow volumes over multiple cardiac time frames and respiratory states [30, 31]. Previous work has shown that 5D flow can be used to detect respiration‐driven flow changes in both healthy volunteers and patients with CHD, showing up to 60% respiratory‐driven variation in CHD venous return [27]. In this study, we employed 5D flow MRI to measure flow energetics over the respiratory cycle. We hypothesized that respiration alters right heart flow energetics and efficiency in Fontan and shunt patients, correlating with cardiac function measures including stroke volume, Fontan flow balance to the left and right PAs, and shunt Qp/Qs. Respiratory‐gated 5D flow introduces variable acceleration across reconstructed respiratory states based on individual respiratory patterns. To ensure observed changes in flow energetics reflect physiology rather than acceleration effects, we also assessed the sensitivity of these metrics to within‐subject variations in 5D flow CS acceleration factors.

## 2 Methods

### 2.1 Study Cohort and Demographics

This IRB‐approved study included 14 Fontan patients (21 ± 8 years, 8 female) and 10 patients with left‐to‐right intracardiac shunts (19 ± 13 years, 8 female), who were prospectively recruited to undergo standard of care cardiothoracic MRI including 2D cine, 2D phase‐contrast, and 4D flow without the use of general anesthesia. 5D flow MRI was acquired at the end of the clinical scans with an additional scan time of 8 min. Patients received ferumoxytol as an off‐label contrast agent prior to scanning, with the exception of one patient who received a gadolinium‐based contrast agent. Two Fontan patients had susceptibility artifacts that led to poor visualization of the aorta, and only the Fontan vessels were included for these patients, with the aorta excluded from group analysis. Patient demographics are summarized in Table 1. The following clinically reported measures of cardiac function were extracted from patients' radiology reports and included in this study: Fontan left–right flow differential (% difference between LPA and RPA net flow) and shunt fraction Qp/Qs based on 2D phase‐contrast or 4D flow net flow ratios, and ventricular stroke volumes based on short‐axis cine imaging. Stroke volume (SV) measurements were indexed to body surface area (BSA).

**TABLE 1:** Cohort demographics and scan parameters.

	Controls (*N* = 9)	Fontan (*N* = 14)	Shunt (*N* = 10)
Age	26 ± 6 years	21 ± 8 years	19 ± 13 years
Sex	1 Female (11%)	8 Female (57%)	8 Female (80%)
		*Combined stroke volume/BSA*: 57 ± 12 mL/m^3^	*LV stroke volume/BSA*: 46 ± 6 mL/m^3^
		*LPA‐RPA flow differential*: 15 ± 12%	*Shunt Qp/Qs*: 1.4 ± 0.6%
		*Single ventricle pathology*: 4 Tricuspid Atresia 4 HLHS 2 DORV 1 Mitral Atresia 1 Pulmonary Atresia 1 Criss‐Cross 1 AVSD	*Shunt pathology*: 4 ASD 4 SVASD 1 VSD 1 SVASD+PAPVR
TR	4.7 ms	4.7–7.1 ms	5.1–5.7 ms
TE	2.9–3.0 ms	2.9–4.4 ms	3.1–3.8 ms
Spatial Res.	2.3 × 2.3 × 2.3–2.5 × 2.5 × 2.5 mm^3^	1.6 × 1.6 × 1.6–2.3 × 2.3 × 2.3 mm^3^	1.7 × 1.7 × 1.7–2.1 × 2.1 × 2.1 mm^3^
Temporal Res.	40–50 ms	40–50 ms	40–50 ms
Flip angle	7–17 degrees	15–25 degrees	15–25 degrees
Venc	150–190 cm/s	100–150 cm/s	150–175 cm/s
Contrast agent	Non‐contrast (*N* = 8) Ferumoxytol (*N* = 1)	Ferumoxytol (*N* = 13) Gadolinium (*N* = 1)	Ferumoxytol (*N* = 10)

Nine age‐appropriate healthy controls (age 26 ± 6 years, 1 female) without CHD were recruited with IRB approval and informed consent and underwent a research MRI scan, including free‐breathing 5D flow. One control scan was performed with ferumoxytol, while the rest received non‐contrast 5D flow imaging.

### 2.2 5D Flow MRI Acquisition

**FIGURE 1:**
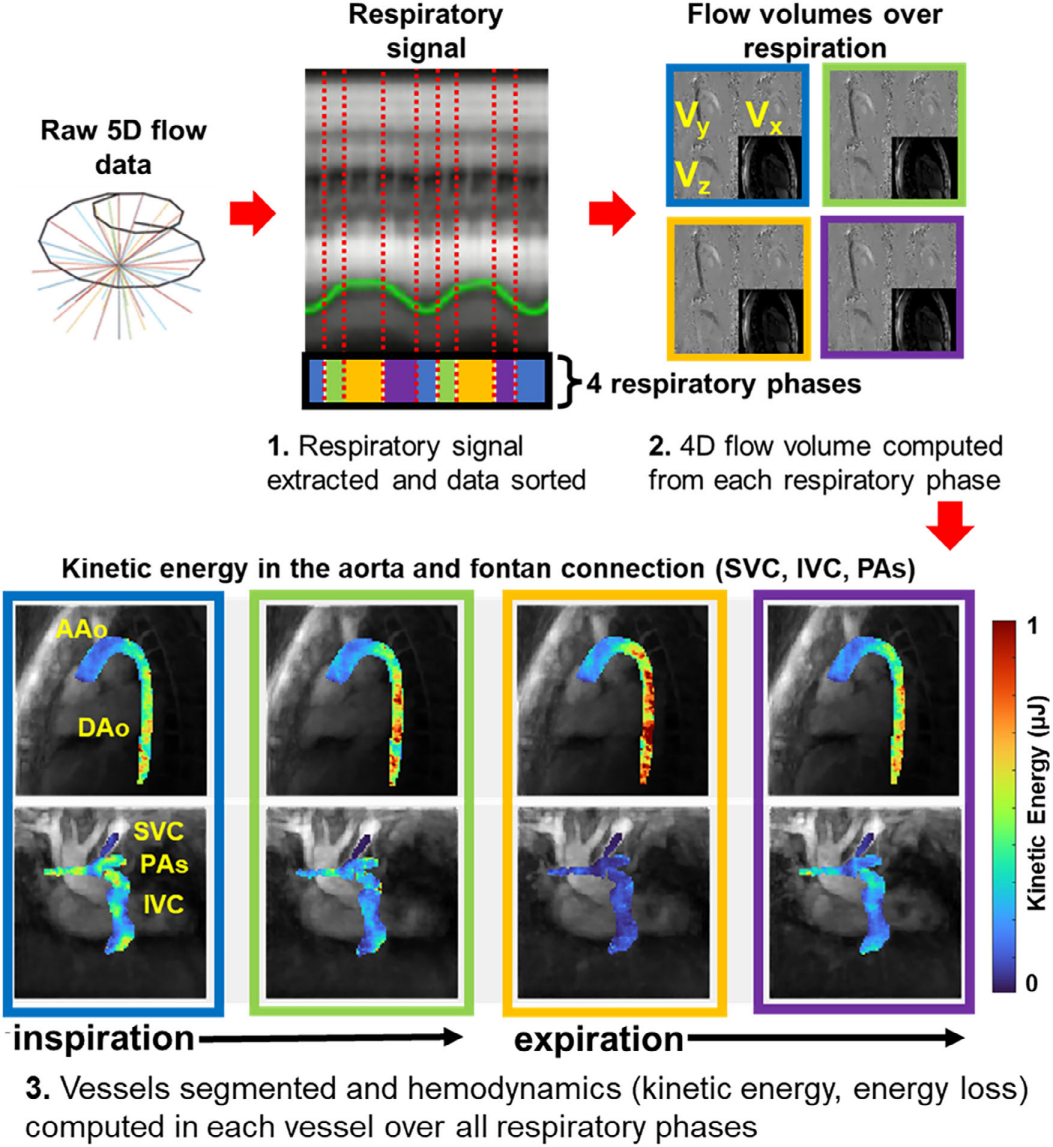
Methods. 5D flow is a free‐running, free‐breathing self‐gating sequence that uses 3D radial k‐space sampling. (1) An interleaved self‐gating projection is used to extract the cardiac and respiratory principal components. (2) 5D flow k‐space lines are sorted to cardiac time frames and respiratory states, and compressed sensing reconstruction is performed to obtain 4D‐flow‐like data (vx, vy, vz, magnitude) over four respiratory states. (3) In each subject, aorta, SVC, IVC, and PAs were segmented. Mean kinetic energy (KE_mean_), total energy loss (EL_total_), and energy loss fraction (EL_total_/KE_mean_) were computed over the cardiac cycle in each vessel for each respiratory state. Example kinetic energy fields are shown for a 28‐year‐old male SVD patient with Fontan reconstruction following mitral atresia.

All subjects underwent free‐running, free‐breathing 5D flow MRI with 3D radial spiral phyllotaxis k‐space sampling and inherent cardiac and respiratory self‐gating using an interleaved super‐inferior projection throughout the acquisition as described previously [29, 30, 31]. The respiratory component of the self‐gating signal was divided into four physiological respiratory states, and k‐space lines were sorted into each state accordingly [27]. Two states were centered on end‐expiration and end‐inspiration, and the remaining k‐space lines were sorted into active expiration and active inspiration, leading to four physiological respiratory states with varying k‐space sub‐sampling acceleration factor (R) based on each subject's physiology. Protocol parameters are summarized in Table 1. 5D flow image reconstruction was performed using compressed sensing (CS) with spatial and temporal regularization across both cardiac and respiratory dimensions [29, 30, 31]. A 3D extension of the RING trajectory correction was used to correct k‐space positions during reconstruction [32]. Cardiac time‐resolved 3D magnitude and flow volumes were generated for each of the four respiratory states (Figure 1).

### 2.3 5D Flow MRI Pre‐Processing and 3D Segmentation

Each 5D flow data set underwent in‐house preprocessing programmed in Matlab (version 2023b, Mathworks) including background phase correction for eddy current effects using identical static tissue segmentations between respiratory states, as well as velocity anti‐aliasing. In each subject, the aorta, pulmonary arteries (PAs), superior vena cava (SVC), and inferior vena cava (IVC) were manually segmented (3D Slicer [33]) using a static phase‐contrast MRA volume derived from the end‐expiration flow and magnitude data. PA segmentation was terminated at the pulmonary branches, SVC segmentation was terminated at the branch point of the right SVC, and IVC segmentation was terminated at the level of the portal veins. End‐expiration 3D segmentations were registered to all other respiratory states with a rigid translation and rotation with linear interpolation implemented using the 3D Slicer BRAINS package [33, 34]. To avoid quantification at the vessel walls while preserving vessel shape, segmentations were first smoothed using a Gaussian kernel with a radius of one voxel (3D Slicer), then eroded using a spherical structural element with a radius of one voxel (Matlab).

### 2.4 Hemodynamic Analysis

In each segmented vessel, voxelwise KE was computed as follows:

$$KE_{\mathrm{vox}}=\frac{1}{2}\rho Vu^{2}$$

where ρ = 1.025 kg/m [3] is the blood density, V is the voxel volume, and u is the measured velocity. Mean KE was calculated as the sum of voxelwise KE over the volume (N voxels) averaged over the cardiac cycle (T phases) and normalized to the volume of the vessel

$$KE_{\text{mean}}=\frac{1}{\mathrm{NVT}}\sum_{n=1}^{N}\sum_{t=1}^{T}KE_{\mathrm{vox}}$$

Voxelwise viscous dissipation rate was computed from the Navier–Stokes energy equation using centered finite differences as follows:

$$\Phi =\frac{1}{2}\sum_{i}\;\sum_{j}{\left[\left(\frac{\partial u_{i}}{\partial x_{i}}+\frac{\partial u_{j}}{\partial x_{j}}\right)-\frac{2}{3}\left(\nabla \cdot u\right)\delta_{\mathrm{ij}}\right]}^{2}$$

where i and j are the principal velocity directions. The voxelwise rate of energy loss was computed as

$${\dot{\mathrm{EL}}}_{\mathrm{vox}}=\mu \Phi V$$

With μ = 0.004 Pas. Total viscous EL per voxel was computed as the temporal integral of EL rate over the cardiac cycle. Total viscous EL per segment was computed as the sum of voxelwise total EL over the volume, normalized to vessel volume [11, 12, 13].

$$EL_{\text{total}}=\frac{1}{\mathrm{NV}}\sum_{n=1}^{N}\int_{0}^{T}{\dot{\mathrm{EL}}}_{\mathrm{vox}}\mathrm{dt}$$

As a metric of flow inefficiency, EL fraction was computed as the ratio of EL_total_/KE_mean_.

Mean values over the respiratory cycle of KE_mean_, EL_total_, and EL fraction were computed and compared between patient groups and controls. The variability in each metric across the four reconstructed respiratory states was computed as (max − min)/mean × 100%. To quantify the impact of respiration on flow energetics, relative changes were computed as the percentage difference in KE_mean_, EL_total_, and EL fraction in each respiratory state from the mean over the four reconstructed respiratory states in each subject.

### 2.5 Sensitivity to Varying CS Acceleration Factors

Reconstructing 5D flow MRI data with four respiratory states resulted in different acceleration factors (R) in each respiratory state, with the highest R during inspiration and shortest R in expiration or end‐expiration. For each respiratory state, R was defined as the Nyquist sampling limit divided by the average number of k‐space lines in each cardiac time frame. To assess the impact of varying acceleration factors R across respiratory states, a second ‘over‐accelerated’ CS reconstruction was performed using the highest (inspiratory) R for all respiratory states. The other three respiratory states were retrospectively down‐sampled in k‐space during reconstruction. To assess the uncertainty in 5D flow measures due to varying acceleration factors, we computed the percent error in over‐accelerated end‐expiratory KE_mean_ and EL_total_ relative to the original end‐expiratory 5D flow data. Changes in the dynamics of relative KE_mean_ and EL_total_ over the respiratory cycle were also assessed.

### 2.6 Sensitivity to Segmentation Boundary

To investigate the contribution of vessel boundary gradients to our respiratory‐resolved measurements, we systematically eroded and dilated each 3D segmentation mask by one voxel and two voxels and recomputed mean kinetic energy and total energy loss in each modulated segmentation. Changes in the dynamics of relative KE_mean_ and EL_total_ over the respiratory cycle were assessed. The results of this analysis in the control cohort are included in the Supporting Information.

### 2.7 Statistics

Comparisons of absolute KE_mean_, EL_total_, and EL fraction between patient groups and controls were evaluated for statistical significance using ANOVA or Kruskal‐Wallis tests, followed by *t*‐test or Wilcoxon rank‐sum with Bonferroni corrections, depending on the normality of the data (Shapiro‐Wilks test).

To test for significant differences in respiratory‐driven dynamics, repeated‐measures ANOVA analysis was performed on the relative measures of KE_mean_, EL_total_, and EL fraction. Independent analyses were performed with each metric in each vessel as the outcome variable. Respiratory state was used as the within‐subject predictor and cohort was used as the between‐subject predictor. Significant alteration in respiratory‐driven dynamics between cohorts was determined using the significance of the interaction term, that is, significant variation in energetics dependent on both cohort and respiratory state. If this interaction was found to be significant, Tukey–Kramer post hoc analysis was performed to evaluate significant differences between cohorts in each respiratory state.

Spearman correlation analysis was performed to test for associations of KE_mean_, EL_total_, and EL fraction in each respiratory state with clinically reported markers of flow alteration severity (Fontan left–right flow differential or shunt Qp/Qs ratio) and left ventricular SV in shunt patients or combined ventricular SV in Fontan patients. We also tested the associations of respiratory variability in KE, EL, and EL fraction with each clinical parameter.

## 3 Results

5D flow MRI with respiratory‐resolved reconstruction was performed successfully in *N* = 24 CHD patients and *N* = 9 controls with CS acceleration factors in each respiratory state ranging from minimum *R* = 37 ± 9 and maximum *R* = 93 ± 84.

### 3.1 Flow Energetics

#### 3.1.1 Fontan and Shunt Patients vs Controls

Compared to controls, Fontan KE_mean_ was decreased by 20% to 62% (SVC *p* < 0.001, IVC *p* < 0.05, PAs *p* < 0.05, Table 2). In addition, Fontan EL_total_ decreased by 20% in the SVC (*p* < 0.05) and 50% in the IVC (*p* < 0.01), resulting in increased SVC and PA EL fraction by 130% and 64% (*p* < 0.01–0.05) versus controls, indicating a marked increase in flow inefficiency. In shunt patients, average KE_mean_ was 89% higher in the PAs (*p* < 0.01) and 57% higher in the aorta (*p* < 0.05). EL_total_ was increased by 186% in the PAs (*p* < 0.01).

**TABLE 2:** Absolute values of mean kinetic energy, total energy loss, and EL fraction.

		SVC	IVC	Pas	Aorta
Control	KE (mJ/L)	21 ± 10	33 ± 8	35 ± 11	46 ± 19
	EL (mJ/L)	5 ± 2	10 ± 5	7 ± 2	9 ± 3
	EL fraction	0.27 ± 0.11	0.30 ± 0.13	0.22 ± 0.06	0.19 ± 0.05
Fontan	KE (mJ/L)	8 ± 6***	28 ± 27*	28 ± 22*	47 ± 20
	EL (mJ/L)	3 ± 2*	5 ± 3**	8 ± 3	9 ± 4
	EL fraction	0.62 ± 0.54**	0.25 ± 0.13	0.36 ± 0.18*	0.21 ± 0.11
Shunt	KE (mJ/L)	31 ± 13	41 ± 21	66 ± 35*	74 ± 24*
	EL (mJ/L)	8 ± 4	12 ± 6	15 ± 7**	13 ± 6
	EL fraction	0.27 ± 0.13	0.33 ± 0.18	0.27 ± 0.13	0.18 ± 0.07

*Note*: *p < 0.05, **p < 0.01, **p < 0.001.

### 3.2 Respiratory‐Driven Alterations in Flow Energetics in Fontan and Shunt Patients

**FIGURE 2:**
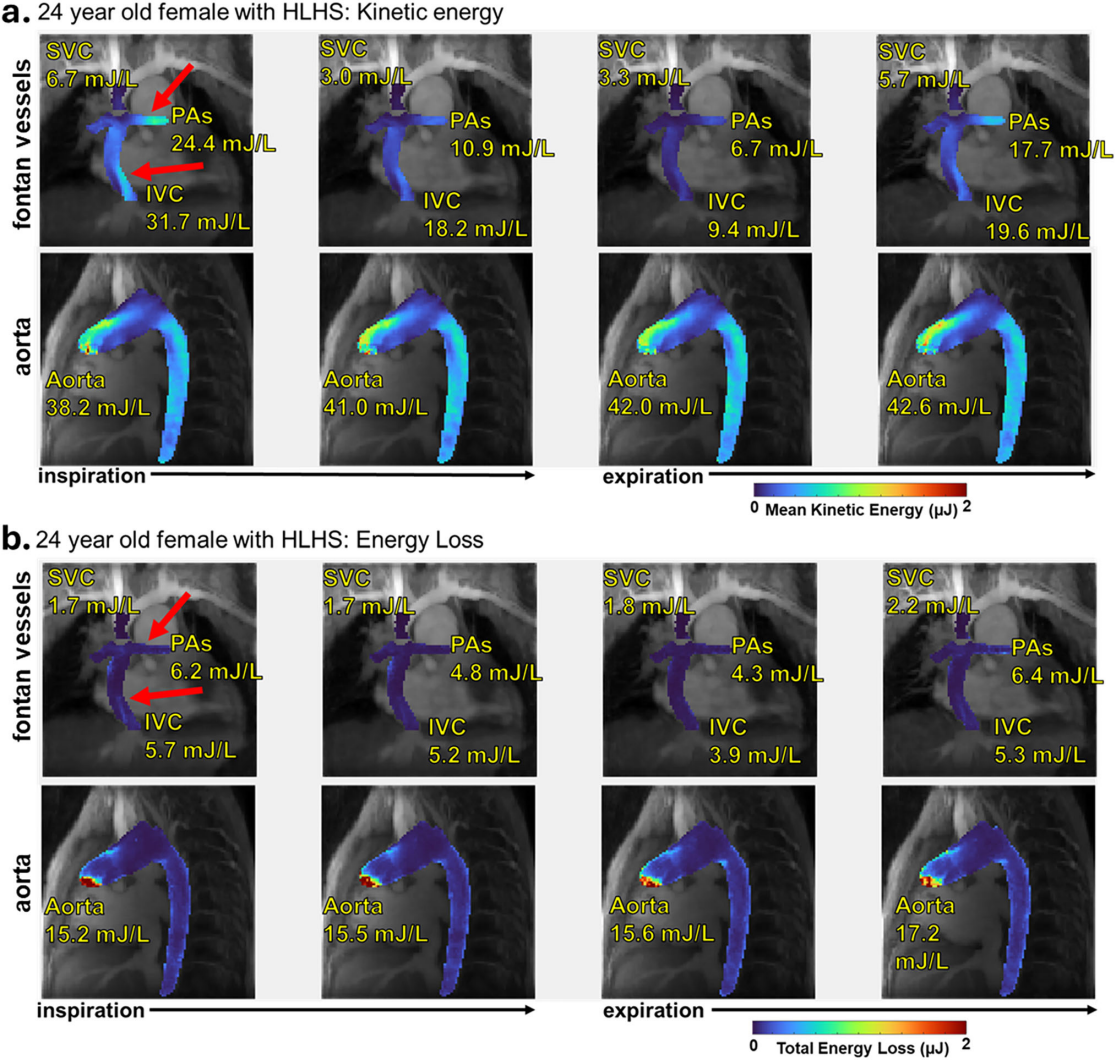
Example of SVD Fontan patient (24‐year‐old female with HLHS) flow energetics over the respiratory cycle in the Fontan connection (a, b, top rows) and thoracic aorta (a, b, bottom rows). The respiratory dimension is displayed left to right from inspiration to end‐expiration. (a) Mean kinetic energy in the IVC and PAs (top row, red arrows) is elevated during inspiration. (b) Total energy loss in the same vessels is not proportionally elevated.

**FIGURE 3:**
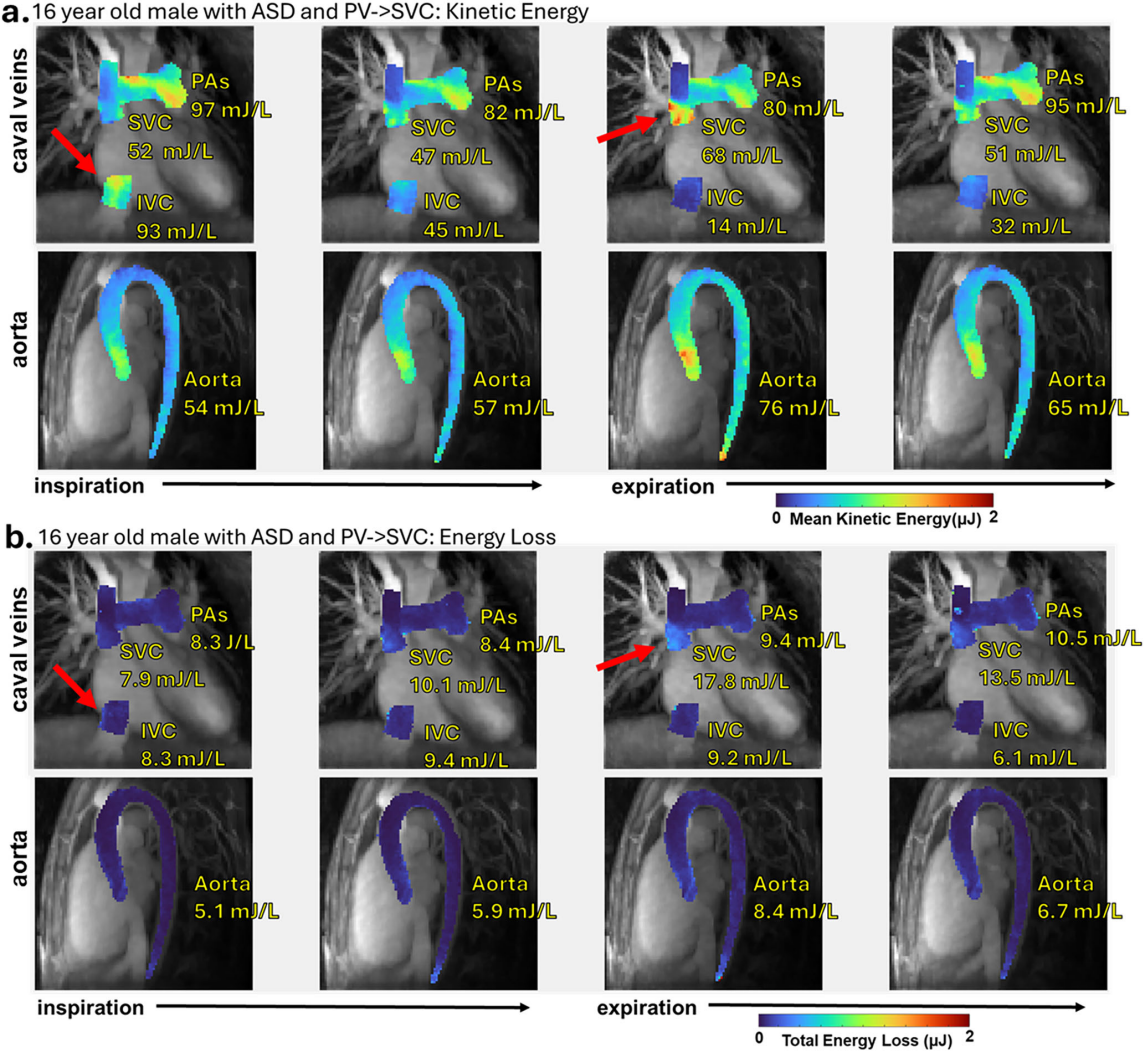
Example of intracardiac shunt patient (16‐year‐old male with atrial septal defect and extracardiac shunt from PVs to SVC) flow energetics over the respiratory cycle in the IVC, SVC and PA (a, b, top rows) and thoracic aorta (a, b, bottom rows). The respiratory dimension is displayed left to right from inspiration to end‐expiration. (a) Mean kinetic energy in the IVC (top row, left red arrow) is elevated during inspiration and kinetic energy in the SVC (top row, right red arrow) is elevated during expiration. (b) Total energy loss in the SVC is proportionally elevated but energy loss in the IVC is not.

**FIGURE 4:**
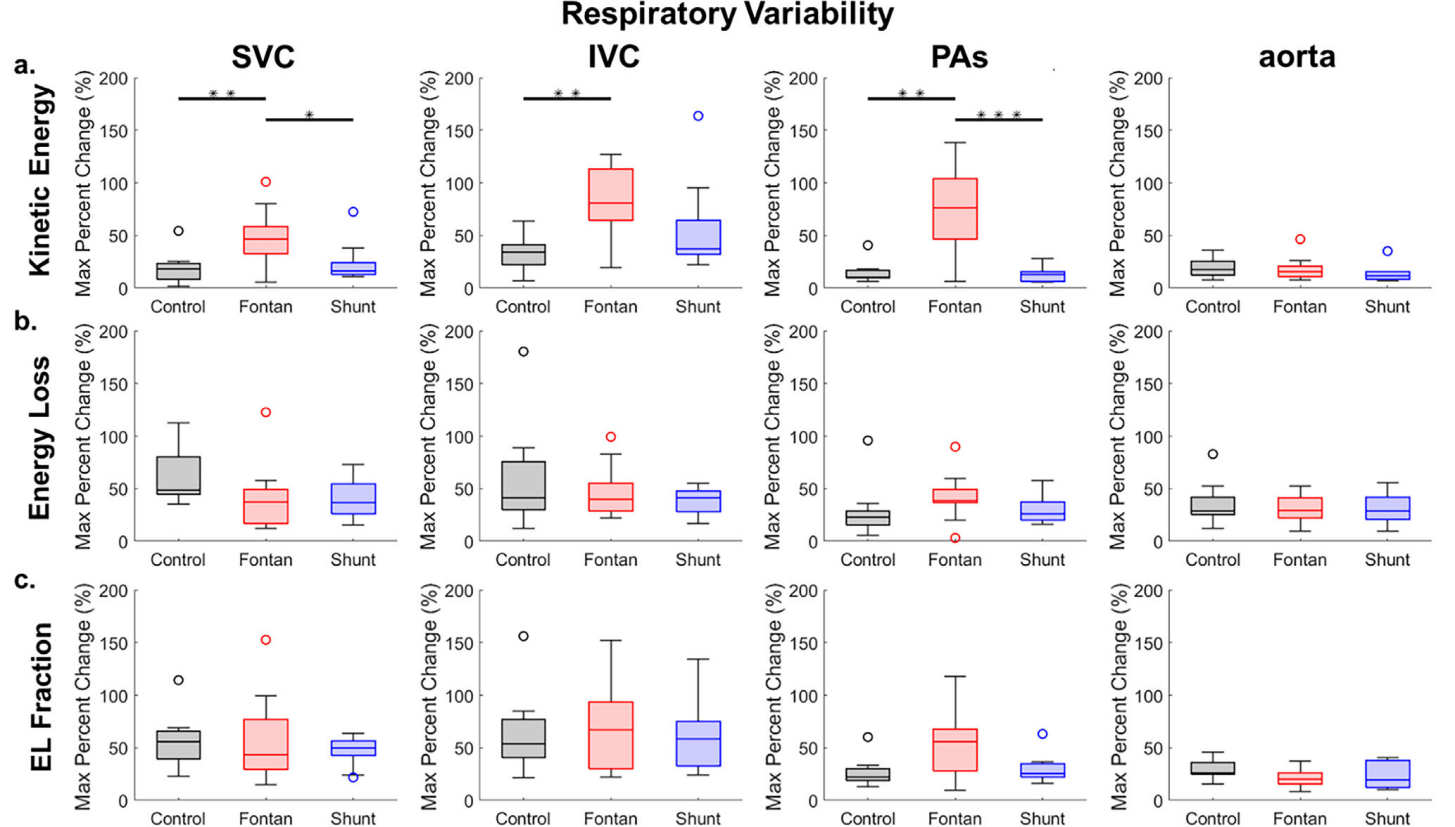
Respiratory Variability of flow energetics. The maximum percent change over the respiratory cycle of each metric was computed for each subject, and box charts for each group are displayed. Significant differences between CHD patient cohorts and controls (gray) were computed with significance denoted (*p < 0.05, **p < 0.01, ***p < 0.001). (a) Respiratory variability of KE_mean_ was significantly elevated in Fontan patients (red) in the SVC, IVC, and PAs compared to controls. Shunt respiratory variability in KE_mean_ (blue) was significantly lower than Fontan patients in the SVC and PAs. (b) Respiratory variability of EL_total_ and (c) EL fraction was not significantly different in patients compared to controls.

Both Fontan and shunt patients demonstrated respiratory‐driven variations in KE_mean_ and EL_total_ over the respiratory cycle. In a representative Fontan patient, KE_mean_ in the PAs and IVC increased during active inspiration (24–32 mJ/L, Figure 2a) compared to expiration (7–9 mJ/L), consistent with enhanced flow from decreased thoracic pressure. EL_total_ in these vessels showed smaller changes during active inspiration (6.2–5.7 mJ/L, Figure 2b) versus expiration (4.3–3.9 mJ/L), suggesting greater dissipation of kinetic energy during expiration. In a shunt patient with a history of atrial septal defect and an extracardiac shunt (pulmonary vein drains to SVC), KE_mean_ increased in the SVC during active expiration (68 vs. 52 mJ/L, Figure 3a) and in the IVC during active inspiration (93 vs. 14 mJ/L), with corresponding EL_total_ increases in the SVC (17.8 vs. 7.9 mJ/L, Figure 3b) but not the IVC (9.2 vs. 8.3 mJ/L), indicating vessel‐specific differences in flow efficiency across respiration. Overall, as shown in Figure 4a, Fontan patients had significantly increased respiratory variability in KE_mean_ by > 2× in the SVC and IVC and > 7× in the PAs compared to both controls (*p* < 0.01) and shunt patients (*p* < 0.001–0.05).

**FIGURE 5:**
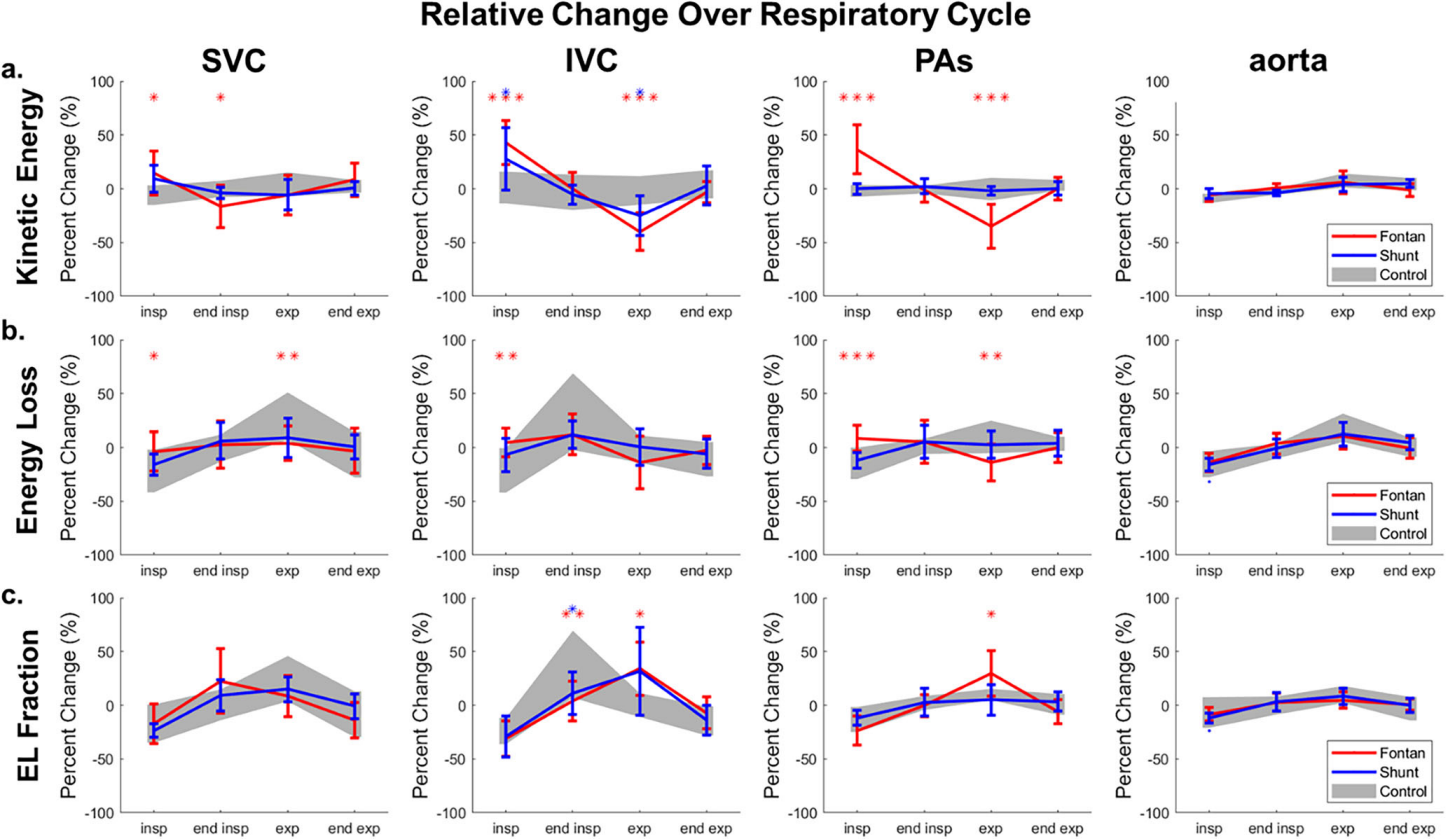
Relative change over the respiratory cycle of flow energetics. The percent change from the mean over respiration in each respiratory state was computed for each metric for each subject and mean and standard deviation are displayed for each group. Two‐way repeated‐measures ANOVA analysis was performed followed by Tukey tests to test the differences in respiratory dynamics between each patient group and controls (gray). Significant differences between patients and controls in each respiratory state are denoted (*p < 0.05, **p < 0.01, ***p < 0.001). (a) Respiratory‐driven dynamics of KE_mean_ were significantly altered in the IVC and PAs of SVD Fontan patients (red) and shunt patients (blue) in the IVC. (b) Respiratory‐driven dynamics of EL_total_ were significantly altered in the SVC, IVC, and PAs of Fontan patients. (c) Respiratory‐driven dynamics of EL fraction were significantly altered in the IVC and PAs of Fontan patients and in the IVC of shunt patients.

Compared to controls, Fontan KE_mean_ increased in the SVC, IVC, and PAs during inspiration (+14% to +43%, *p* < 0.05, Figure 5a), decreased in the SVC at end‐inspiration (−17%, *p* < 0.05), and decreased in the IVC and PAs during active expiration (−40%, −35%, *p* < 0.001). EL_total_ increased in the SVC, IVC, and PAs during active inspiration (−3% to 9% vs. −20% to −11%, *p* < 0.05, Figure 5b) and decreased in the SVC and PAs during expiration (4%, −14% vs. 29%, 10%, *p* < 0.01). Consequently, EL fraction decreased in the IVC at end‐inspiration (+4% vs. +38% *p* < 0.01, Figure 5c) and increased in the IVC and PAs during active expiration (+34%, +30%, *p* < 0.05).

In shunt patients, IVC KE_mean_ decreased during active inspiration (+28%, *p* < 0.05, Figure 5a) and decreased during active expiration (−25%, *p* < 0.05). With unaltered relative EL_total_ (Figure 5b), shunt end‐inspiratory IVC EL fraction decreased compared to controls (+11% vs. +38%, *p* < 0.05, Figure 5c).

### 3.3 Fontan Patients: Correlation of Flow Energetics With SV and Fontan Left–Right Flow Difference

In the SVC, we found a significant positive relationship between increased EL_total_ over multiple respiratory states and higher Fontan left–right flow differential (ρ = 0.77–0.79, *p* < 0.01, Table S1). Ventricular SV was positively associated with increased expiratory IVC EL_total_ (ρ = −0.58, *p* < 0.05, Table S1) and aorta EL_total_ (ρ = 0.68–0.74, *p* < 0.05, Table S1) and negatively correlated with increased respiratory variability in PA EL_total_ (ρ = 0.57, *p* < 0.05, Table S1). There was a significant negative correlation between expiratory IVC EL_total_ and Fontan left–right flow differential (ρ = −0.66, *p* < 0.05, Table S2). These results indicate that reduced cardiac function was related to respiratory‐driven changes in flow efficiency in the vessels of the Fontan connection.

### 3.4 Shunt Patients: Correlations of Respiration‐Resolved Flow Energetics With SV and Q_p_/Q_s_ Ratio

Correlation analysis revealed a significant negative relationship between increased respiratory variability in SVC KE_mean_ and reduced SV (ρ = −0.73, *p* < 0.05, Table S3) and a positive correlation with increased Qp/Qs (ρ = 0.64, *p* < 0.05, Table S3). There was a significant negative correlation between decreased expiratory KE_mean_ in the IVC and increased Qp/Qs (ρ = −0.70, *p* < 0.05, Table S4). These results indicate that intracardiac flow shunted from left‐to‐right is related to variability in venous return that is modulated by respiration.

### 3.5 Impact of Varying Acceleration Factors on Respiratory‐Resolved Flow Energetics

**FIGURE 6:**
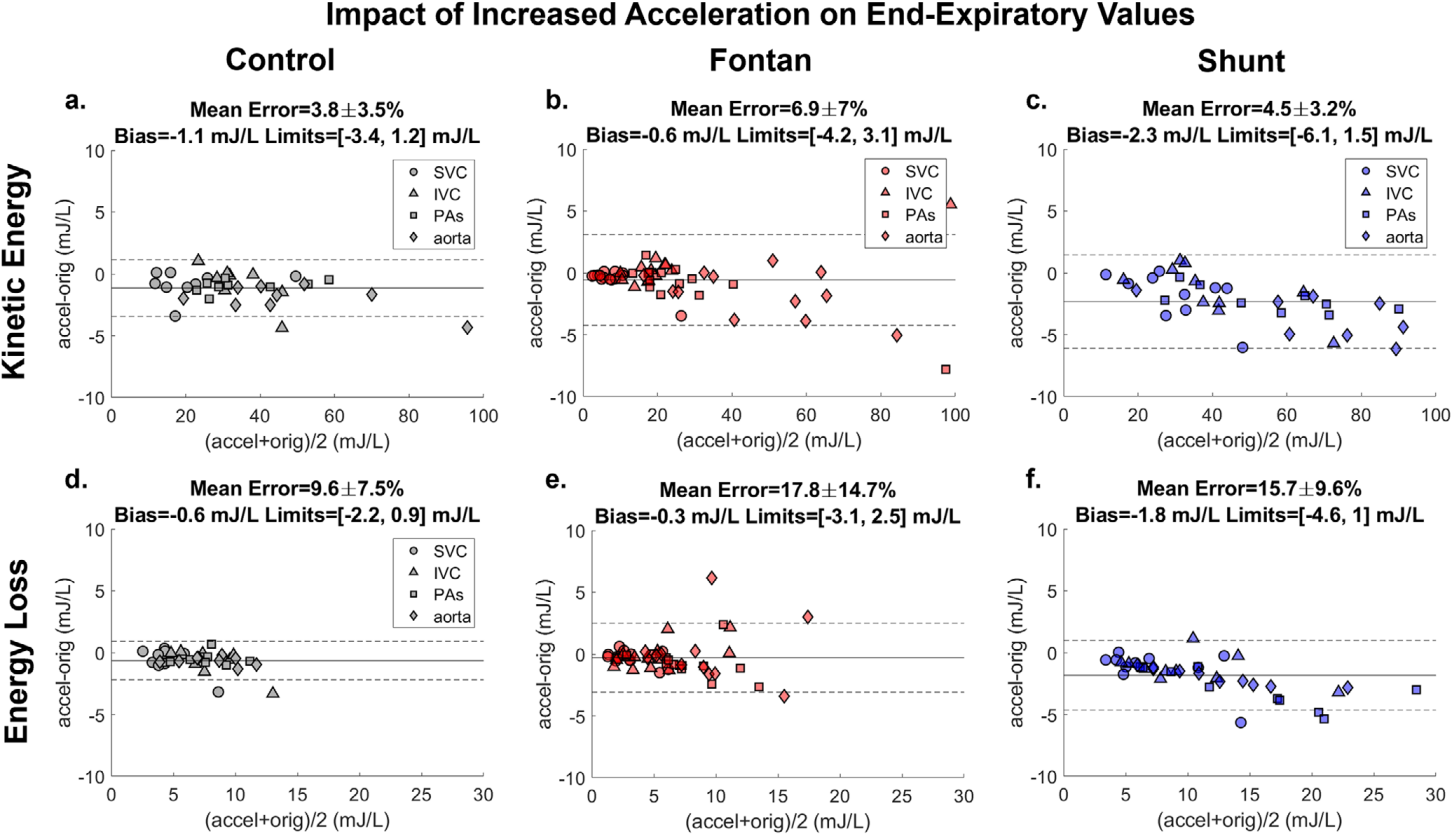
Impact of Increased Acceleration on End‐Expiratory values of flow energetics. To test the impact of varying acceleration over the respiratory cycle, we performed 5D flow reconstruction with the highest amount of per‐subject acceleration (inspiration) applied to all respiratory states. We evaluated the agreement of the accelerated data compared to the original data using the end‐expiration respiratory state, which had the greatest change in acceleration in this experiment. We performed Bland–Altman analysis for KE_mean_ measured in all vessels in (a) controls, (b) Fontan patients, and (c) shunt patients. We also evaluated agreement in EL_total_ (d–f).

Over‐accelerated CS 5D flow matching the highest R per subject (*R* = 93 ± 84) showed low limits of agreement with the acquired 5D flow data for end‐expiration KE_mean_ across cohorts (< ±4 mJ/L, Figure 6a–c). KE_mean_ was significantly underestimated (−2.3 to −0.06 mJ/L, *p* < 0.001), with the largest underestimation in Fontan patients, yielding a 4%–7% percent error. End‐expiration EL_total_ limits of agreement were also low across cohorts (< ±2 mJ/L, Figure 6d–f), with significant underestimation (−0.3 to −1.8 mJ/L, *p* < 0.001), corresponding to a 9%–18% error.

In controls, SVC and IVC respiratory changes were preserved, while aorta KE_mean_, aorta EL_total_, and PA EL_total_ were altered with higher CS acceleration decreasing respiratory variability (*p* < 0.05, Figures S1a, S2a). In Fontan patients, KE_mean_ and EL_total_ dynamics remained unchanged by increased CS acceleration (Figures S1b, S2b). Shunt patients showed a similar decrease in respiratory variability compared to controls (*p* < 0.01, Figures S1c, S2c). Changes in flow energetics due to over‐acceleration were < 10% in any respiratory state. Significant findings between cohorts reported above showed substantially higher changes in relative KE_mean_ and EL_total_—exceeding 2× the error due to over acceleration.

### 3.6 Sensitivity to Segmentation Vessel Boundary

Erosion and dilation of segmentations did not significantly impact the assessment of respiration‐driven relative KE_mean_ and EL_total_ dynamics over the respiratory cycle (Figures S3–S4). Further discussion of this result can be found in the Supporting Information.

## 4 Discussion

In this study, we characterized changes in 3D blood flow energetics over the respiratory cycle using 5D flow MRI. The main findings of this study are (1) Fontan and shunt patients demonstrated altered flow energetics in pulmonary vessels compared to controls, (2) the dynamic profile of changes in flow energetics over the respiratory cycle differed from controls, (3) increased chest pressure during expiration led to decreased KE_mean_ of the IVC venous return compared to inspiration in both patient groups, which was paired with an increase in flow inefficiency (EL fraction) during expiration, (4) Fontan patients showed respiratory‐driven SVC, IVC, and PA flow energetics which were associated with altered SV and Fontan left–right flow differential, and (5) expiratory IVC flow energetics were correlated with shunt Qp/Qs. These findings indicate the importance of considering the respiratory impacts on venous returns and PA flow when evaluating cardiac function in CHD patients.

Our study showed an overall decrease in KE_mean_ and EL_total_ in the right heart vessels of Fontan patients, but an increase in the EL fraction compared to controls. This finding is consistent with previous studies, which have shown decreased KE_mean_ in the single ventricle and the Fontan connection using 4D flow [15, 16, 19, 20, 35]. Prior work has focused on the single ventricle, but here we directly quantified the amount of energy dissipated to viscous forces through pulmonary circulation [20, 36]. Both EL_total_ and KE_mean_ were indexed to vessel volume and the computed EL fraction captured the proportion of dissipated energy in each vessel for comparison between heterogeneous cohorts. Our results indicate that flow through the Fontan connection is less efficient than control right‐heart anatomy. In left‐to‐right shunt patients, increased KE_mean_ and EL_total_ in the PA indicates that increased flow energy in the pulmonary system is partially dissipated by viscous forces.

Both CHD patient cohorts demonstrated increased respiratory variability in flow energetics, indicating increased sensitivity to changes in chest pressure compared to controls. In both CHD cohorts, the KE_mean_ of IVC venous return increased during active inspiration when chest pressures are lower and decreased during expiration when chest pressures are higher. The lower pulmonary pressure environment in Fontan and shunt patients may enable this chest‐pressure‐driven component of altered systemic venous return. These dynamic differences are consistent with echocardiography and 2D phase‐contrast studies in Fontan patients that have shown greater flow velocities when measured in inspiration compared to expiration [24, 26]. In both CHD patient groups, decreased KE_mean_ during expiration resulted in increased flow inefficiency (EL fraction). This may account for the large respiratory‐driven variability in the amount of work done by the system (KE_mean_), with increased KE_mean_ in inspiration compensating for the increased proportion of energy dissipated during expiration.

In intracardiac shunt patients, respiratory changes in IVC KE_mean_ and EL fraction were not coupled with changes in the PAs. This indicates the role of the right ventricle in decoupling respiratory dynamics between venous return and pulmonary flow. There are multiple possible explanations for this phenomenon. The PAs and aorta may have more consistent forward flow over the respiratory cycle, and the difference in filling versus PA flow may be accounted for by changes in RA or RV ESV and EDV over the respiratory cycle. Additionally, PA flow KE_mean_ was increased at end‐inspiration and decreased at end‐expiration, indicating a delayed effect in response to increased inspiratory venous return. However, PA flow in shunt patients is much faster than IVC flow and small modulations proportional to changes in IVC flow may be difficult to detect. To further study this effect, methods for improved data quality of 5D flow MRI such as dual‐venc imaging are needed.

Our results showed that flow efficiency during different states of the respiratory cycle had varying association with markers of altered flow (left–right PA flow differential in Fontan, Qp/Qs in shunt patients). Uneven PA flow distribution is related to poor exercise tolerance in Fontan patients and may be caused by downstream pressure differences or vascular geometry [37, 38]. Our results indicate that the mechanisms of altered left–right PA flow in Fontan patients may be partially driven by the imbalanced impacts of respiration on upper versus lower venous return. Increased combined ventricular SV was significantly associated with decreased respiratory‐driven variability in PA EL_total_ and increased expiratory SVC and aorta EL_total_. These results indicate that diminished cardiac function may be driven by the limits of venous return during active expiration, where increased aorta flow and decreased pulmonary flow resulted in increased viscous dissipation. Measurements with gating centered at end‐expiration (commonly used for 4D flow and 2D phase‐contrast) may thus underestimate Fontan dysfunction. In shunt patients, more severe shunting (increased Qp/Qs) was associated with the respiratory‐driven reductions in venous return during active expiration. Importantly, these effects cannot be measured using breath‐held or respiratory‐gated flow acquisitions, as the full range of pressure‐driven energetic variation will be underestimated. 5D flow MRI may thus help to better understand these mechanisms and their impact on clinical outcomes in patients with right‐sided CHD.

The results of our sensitivity analysis demonstrated that measures of flow energetics were robust to increased 5D flow CS acceleration factors R. Despite an underestimation error in both KE_mean_ (4%–7%) and EL_total_ (9%–18%), respiratory‐driven dynamics in most vessels were preserved when the maximum acceleration was applied. Error due to varied acceleration was 2‐fold smaller compared to the observed respiratory differences in flow energetic measures between cohorts. The largest impact of CS acceleration was detected in the aorta, where respiratory dynamics became flattened due to increased acceleration (i.e., KE_mean_ and EL_total_ are underestimated and less variability between respiratory states). However, the stability of right‐heart vessel dynamics despite additional acceleration indicates that the respiratory‐driven changes in hemodynamics detected in this study are reflective of patient physiology, as opposed to technical limitations.

### 4.1 Limitations

This study was limited by the small and heterogeneous cohorts of CHD patients. Although there is no significant difference in age between controls and CHD patients in this study, no direct age‐matching was performed and the study findings may still be confounded by age. One potential age‐related confounding variable is respiratory rate, which tends to be higher in younger children [39]. Our previous work indicates that capturing blood flow over four respiratory states is sufficient to detect respiratory‐driven changes in both healthy adults and pediatric congenital heart disease patients [27]. Nonetheless, larger studies of 5D flow in CHD and healthy pediatric populations are needed to untangle the effects of age, respiration, and cardiovascular flow. This is especially important in the case of young children that might be evaluated under general anesthesia, as positive pressure ventilation modulates respiratory‐driven pressure gradients. While all patients in this study were assessed under free‐breathing conditions, initial studies indicate that ventilated anesthesia further modulates respirophasic flow energetic dynamics [40]. Further study into the effects of ventilation and general anesthesia on measured flow in younger CHD patients is warranted.

5D flow was added to the end of CHD patients' routine clinical imaging. While this approach enabled recruitment, a systemic investigation of the impact of 5D flow scan parameters on respiration‐resolved flow energetics was not performed. We did not quantify the effect of changes in spatial or temporal resolution on the measured CHD hemodynamics, as multiple scans with varying parameters were not feasible. Further, CHD patients in this study received ferumoxytol contrast as part of their routine imaging. A majority of the controls included in this study did not receive contrast. It is possible that the enhanced signal in CHD patients' blood tissue decreased velocity noise and improved convergence of the iterative CS reconstruction, limiting comparison to non‐contrast control data. Future studies are warranted to systematically assess the impact of ferumoxytol contrast on 5D flow data quality or flow quantification. Finally, we evaluated the impacts of increased CS acceleration on flow energetic quantification accuracy but did not quantify its impacts on signal or noise. Future studies should address the choice of the venc on respiration‐resolved flow measurements and consider a dual‐velocity encoding approach if velocity noise is found to be a limiting factor in this analysis.

## 5 Conclusions

This study demonstrated that flow energetics and flow efficiency are modulated by respiration in right‐sided CHD patients. Our study demonstrated that capturing the entire range of flow energetic measurements over the respiratory cycle provides novel insights into the mechanisms of Fontan flow abnormalities and intracardiac shunt severity. 5D flow MRI is a powerful tool for capturing the complexities of flow in the low‐pressure environment of right‐sided congenital heart disease and may be useful for improving our understanding of the factors driving adverse outcomes in CHD.

## Funding

This work was supported by the National Institute of Biomedical Imaging and Bioengineering (T32EB025766); National Heart, Lung, and Blood Institute (F30HL165805, F31HL165915, R01HL115828‐09); Schweizerischer Nationalfonds zur Förderung der Wissenschaftlichen Forschung (320030B_201292).

## Supporting information


**Table S1:** Correlations with combined ventricular stroke volume in Fontan patients (*p < 0.05).
**Table S2:** Correlations with LPA‐RPA flow differential in Fontan patients (*p < 0.05, **p < 0.01).
**Table S3:** Correlations with left ventricular stroke volume in shunt patients (*p < 0.05).
**Table S4:** Correlations with Qp/Qs in shunt patients (*p < 0.05).
**Figure S1:** Impact of increased acceleration on respiratory‐driven dynamics of kinetic energy. We computed KEmean over the cardiac cycle in each respiratory state in original and accelerated data. We compared for significant differences in dynamics between paired data using repeated‐measures ANOVA followed by Tukey tests. Respiratory variability (resp var) was computed as the maximum percent change in KEmean over the respiratory cycle and compared between original and accelerated data. Significant differences are denoted (*p < 0.05, **p < 0.01, ***p < 0.001). (a) Respiratory‐driven dynamics in KEmean were significantly different with increased acceleration in the aorta of controls, and respiratory variability was decreased. KEmean dynamics were not significantly altered in (b) Fontan patients or (c) shunt patients.
**Figure S2:** Impact of increased acceleration on respiratory‐driven dynamics of energy loss. We computed ELtotal over the cardiac cycle in each respiratory state in original and accelerated data. We compared for significant differences in dynamics between paired data using repeated‐measures ANOVA followed by Tukey tests. Respiratory variability was computed as the maximum percent change in energy loss over the respiratory cycle and compared between original and accelerated data. Significant differences are denoted (*p < 0.05, **p < 0.01, ***p < 0.001). (a) Respiratory‐driven dynamics in ELtotal were significantly different with increased acceleration in the PAs and aorta of controls, and respiratory variability was significantly decreased in both vessels. (b) ELtotal dynamics were not significantly altered in Fontan patients. (c) Respiratory variability was decreased in the aorta of shunt patients.
**Figure S3:** Impact of segmentation erosion on respiratory‐driven dynamics of kinetic energy and energy loss. In controls, we computed relative changes in KEmean and ELtotal over the cardiac cycle in each respiratory state in original segmentations and segmentations eroded by spherical elements with radius 1 voxel (1×) and 2 voxels (2×). We compared for significant differences in dynamics between paired data using repeated‐measures ANOVA followed by Tukey tests. Respiratory variability (resp var) was computed as the maximum percent change in each metric over the respiratory cycle and compared between original and accelerated data. No significant differences were found in relative (a) KEmean or (b) ELtotal.
**Figure S4:** Impact of segmentation dilation on respiratory‐driven dynamics of kinetic energy and energy loss. In controls, we computed relative changes in KEmean and ELtotal over the cardiac cycle in each respiratory state in original segmentations and segmentations dilated by spherical elements with radius 1 voxel (1×) and 2 voxels (2×). We compared for significant differences in dynamics between paired data using repeated‐measures ANOVA followed by Tukey tests. Respiratory variability (resp var) was computed as the maximum percent change in each metric over the respiratory cycle and compared between original and accelerated data. No significant differences were found in relative (a) KEmean or (b) ELtotal.

## Data Availability

Research data are not shared.

## References

[1] P. Beerbaum , H. Körperich , P. Barth , H. Esdorn , J. Gieseke , and H. Meyer , “Noninvasive Quantification of Left‐To‐Right Shunt in Pediatric Patients: Phase‐Contrast Cine Magnetic Resonance Imaging Compared With Invasive Oximetry,” Circulation 103, no. 20 (2001): 2476–2482, 10.1161/01.cir.103.20.2476. 11369688

[2] A. Deri and K. English , “EDUCATIONAL SERIES IN CONGENITAL HEART DISEASE: Echocardiographic Assessment of Left to Right Shunts: Atrial Septal Defect, Ventricular Septal Defect, Atrioventricular Septal Defect, Patent Arterial Duct,” Echo Research and Practice 5, no. 1 (2018): R1–R16, 10.1530/ERP-17-0062. 29432197 PMC5840804

[3] K. Pushparajah , “Non‐Invasive Imaging in the Evaluation of Cardiac Shunts for Interventional Closure,” Frontiers in Cardiovascular Medicine 8 (2021): 651726, 10.3389/fcvm.2021.651726. 34222361 PMC8253251

[4] W. G. Hundley , H. F. Li , R. A. Lange , et al., “Assessment of Left‐To‐Right Intracardiac Shunting by Velocity‐Encoded, Phase‐Difference Magnetic Resonance Imaging. A Comparison With Oximetric and Indicator Dilution Techniques,” Circulation 91, no. 12 (1995): 2955–2960, 10.1161/01.cir.91.12.2955. 7796506

[5] M. Gewillig and S. C. Brown , “The Fontan Circulation After 45 Years: Update in Physiology,” Heart (British Cardiac Society) 102, no. 14 (2016): 1081–1086, 10.1136/heartjnl-2015-307467. 27220691 PMC4941188

[6] J. A. Hauser , A. M. Taylor , and B. Pandya , “How to Image the Adult Patient With Fontan Circulation,” Circulation. Cardiovascular Imaging 10, no. 5 (2017): e004273, 10.1161/CIRCIMAGING.116.004273. 28495823

[7] P. Dyverfeldt , M. Bissell , A. J. Barker , et al., “4D Flow Cardiovascular Magnetic Resonance Consensus Statement,” Journal of Cardiovascular Magnetic Resonance 17, no. 1 (2015): 72, 10.1186/s12968-015-0174-5. 26257141 PMC4530492

[8] M. M. Bissell , F. Raimondi , L. Ait Ali , et al., “4D Flow Cardiovascular Magnetic Resonance Consensus Statement: 2023 Update,” Journal of Cardiovascular Magnetic Resonance 25, no. 1 (2023): 40, 10.1186/s12968-023-00942-z. 37474977 PMC10357639

[9] M. Markl , S. Schnell , and A. J. Barker , “4D Flow Imaging: Current Status to Future Clinical Applications,” Current Cardiology Reports 16, no. 5 (2014): 481, 10.1007/s11886-014-0481-8. 24700368

[10] S. Nordmeyer , E. Riesenkampff , D. Messroghli , et al., “Four‐Dimensional Velocity‐Encoded Magnetic Resonance Imaging Improves Blood Flow Quantification in Patients With Complex Accelerated Flow,” Journal of Magnetic Resonance Imaging 37, no. 1 (2013): 208–216, 10.1002/jmri.23793. 22976284

[11] A. J. Barker , P. van Ooij , K. Bandi , et al., “Viscous Energy Loss in the Presence of Abnormal Aortic Flow,” Magnetic Resonance in Medicine 72, no. 3 (2014): 620–628, 10.1002/mrm.24962. 24122967 PMC4051863

[12] V. P. Kamphuis , J. J. M. Westenberg , R. L. F. van der Palen , et al., “Scan‐Rescan Reproducibility of Diastolic Left Ventricular Kinetic Energy, Viscous Energy Loss and Vorticity Assessment Using 4D Flow MRI: Analysis in Healthy Subjects,” International Journal of Cardiovascular Imaging 34, no. 6 (2018): 905–920, 10.1007/s10554-017-1291-z. 29305740

[13] M. S. M. Elbaz , R. J. van der Geest , E. E. Calkoen , et al., “Assessment of Viscous Energy Loss and the Association With Three‐Dimensional Vortex Ring Formation in Left Ventricular Inflow: In Vivo Evaluation Using Four‐Dimensional Flow MRI,” Magnetic Resonance in Medicine 77, no. 2 (2017): 794–805, 10.1002/mrm.26129. 26924448 PMC5297883

[14] F. M. Rijnberg , J. J. M. Westenberg , H. C. van Assen , et al., “4D Flow Cardiovascular Magnetic Resonance Derived Energetics in the Fontan Circulation Correlate With Exercise Capacity and CMR‐Derived Liver Fibrosis/Congestion,” Journal of Cardiovascular Magnetic Resonance 24, no. 1 (2022): 21, 10.1186/s12968-022-00854-4. 35346249 PMC8962091

[15] V. P. Kamphuis , M. S. M. Elbaz , P. J. van den Boogaard , et al., “Disproportionate Intraventricular Viscous Energy Loss in Fontan Patients: Analysis by 4D Flow MRI,” European Heart Journal Cardiovascular Imaging 20, no. 3 (2019): 323–333, 10.1093/ehjci/jey096. 30060051

[16] P. Sjöberg , E. Heiberg , P. Wingren , et al., “Decreased Diastolic Ventricular Kinetic Energy in Young Patients With Fontan Circulation Demonstrated by Four‐Dimensional Cardiac Magnetic Resonance Imaging,” Pediatric Cardiology 38, no. 4 (2017): 669–680, 10.1007/s00246-016-1565-6. 28184976 PMC5388704

[17] D. R. Rutkowski , G. Barton , C. J. François , H. L. Bartlett , P. V. Anagnostopoulos , and A. Roldán‐Alzate , “Analysis of Cavopulmonary and Cardiac Flow Characteristics in Fontan Patients: Comparison With Healthy Volunteers,” Journal of Magnetic Resonance Imaging 49, no. 6 (2019): 1786–1799, 10.1002/jmri.26583. 30635978 PMC7359045

[18] V. P. Kamphuis , M. S. M. Elbaz , P. J. van den Boogaard , et al., “Stress Increases Intracardiac 4D Flow Cardiovascular Magnetic Resonance ‐Derived Energetics and Vorticity and Relates to VO2max in Fontan Patients,” Journal of Cardiovascular Magnetic Resonance 21, no. 1 (2019): 43, 10.1186/s12968-019-0553-4. 31340834 PMC6657113

[19] V. P. Kamphuis , A. A. W. Roest , P. J. van den Boogaard , et al., “Hemodynamic Interplay of Vorticity, Viscous Energy Loss, and Kinetic Energy From 4D Flow MRI and Link to Cardiac Function in Healthy Subjects and Fontan Patients,” American Journal of Physiology. Heart and Circulatory Physiology 320, no. 4 (2021): H1687–H1698, 10.1152/ajpheart.00806.2020. 33635164

[20] F. M. Rijnberg , J. F. Juffermans , M. G. Hazekamp , et al., “Segmental Assessment of Blood Flow Efficiency in the Total Cavopulmonary Connection Using Four‐Dimensional Flow Magnetic Resonance Imaging: Vortical Flow Is Associated With Increased Viscous Energy Loss Rate,” European Heart Journal Open 1, no. 2 (2021): oeab018, 10.1093/ehjopen/oeab018. 35919267 PMC9241567

[21] J. Rychik , A. M. Atz , D. S. Celermajer , et al., “Evaluation and Management of the Child and Adult With Fontan Circulation: A Scientific Statement From the American Heart Association,” Circulation 140, no. 6 (2019): e234–e284, 10.1161/CIR.0000000000000696. 31256636

[22] P. M. Engelfriet , M. G. J. Duffels , T. Möller , et al., “Pulmonary Arterial Hypertension in Adults Born With a Heart Septal Defect: The Euro Heart Survey on Adult Congenital Heart Disease,” Heart (British Cardiac Society) 93, no. 6 (2007): 682–687, 10.1136/hrt.2006.098848. 17164490 PMC1955187

[23] A. C. M. J. van Riel , M. J. Schuuring , I. D. van Hessen , et al., “Contemporary Prevalence of Pulmonary Arterial Hypertension in Adult Congenital Heart Disease Following the Updated Clinical Classification,” International Journal of Cardiology 174, no. 2 (2014): 299–305, 10.1016/j.ijcard.2014.04.072. 24794056

[24] D. D. Gabbert , C. Hart , M. Jerosch‐Herold , et al., “Heart Beat but Not Respiration Is the Main Driving Force of the Systemic Venous Return in the Fontan Circulation,” Scientific Reports 9, no. 1 (2019): 2034, 10.1038/s41598-019-38848-5. 30765829 PMC6376003

[25] K. S. Ha , J. Y. Choi , S. Y. Jung , and H. K. Park , “Characterization of Flow Efficiency, Pulsatility, and Respiratory Variability in Different Types of Fontan Circuits Using Quantitative Parameters,” Yonsei Medical Journal 60, no. 1 (2019): 56–64, 10.3349/ymj.2019.60.1.56. 30554491 PMC6298895

[26] Y. Yamasaki , S. Kawanami , T. Kamitani , et al., “Noninvasive Quantification of Left‐To‐Right Shunt by Phase Contrast Magnetic Resonance Imaging in Secundum Atrial Septal Defect: The Effects of Breath Holding and Comparison With Invasive Oximetry,” International Journal of Cardiovascular Imaging 34, no. 6 (2018): 931–937, 10.1007/s10554-018-1297-1. 29340831

[27] E. K. Weiss , J. Baraboo , C. K. Rigsby , et al., “Respiratory‐Resolved Five‐Dimensional Flow Cardiovascular Magnetic Resonance : In‐Vivo Validation and Respiratory‐Dependent Flow Changes in Healthy Volunteers and Patients With Congenital Heart Disease,” Journal of Cardiovascular Magnetic Resonance 26, no. 2 (2024): 101077, 10.1016/j.jocmr.2024.101077. 39098573 PMC11417305

[28] P. Dyverfeldt and T. Ebbers , “Comparison of Respiratory Motion Suppression Techniques for 4D Flow MRI,” Magnetic Resonance in Medicine 78, no. 5 (2017): 1877–1882, 10.1002/mrm.26574. 28074541 PMC6084364

[29] L. E. Ma , J. Yerly , D. Piccini , et al., “5D Flow MRI: A Fully Self‐Gated, Free‐Running Framework for Cardiac and Respiratory Motion‐Resolved 3D Hemodynamics,” Radiology: Cardiothoracic Imaging 2, no. 6 (2020): e200219, 10.1148/ryct.2020200219. 33385164 PMC7755133

[30] L. Feng , S. Coppo , D. Piccini , et al., “5D Whole‐Heart Sparse MRI,” Magnetic Resonance in Medicine 79, no. 2 (2018): 826–838, 10.1002/mrm.26745. 28497486 PMC5681898

[31] L. Di Sopra , D. Piccini , S. Coppo , M. Stuber , and J. Yerly , “An Automated Approach to Fully Self‐Gated Free‐Running Cardiac and Respiratory Motion‐Resolved 5D Whole‐Heart MRI,” Magnetic Resonance in Medicine 82, no. 6 (2019): 2118–2132, 10.1002/mrm.27898. 31321816

[32] S. Rosenzweig , H. C. M. Holme , and M. Uecker , “Simple Auto‐Calibrated Gradient Delay Estimation From Few Spokes Using Radial Intersections (RING),” Magnetic Resonance in Medicine 81, no. 3 (2019): 1898–1906, 10.1002/mrm.27506. 30489652

[33] A. Fedorov , R. Beichel , J. Kalpathy‐Cramer , et al., “3D Slicer as an Image Computing Platform for the Quantitative Imaging Network,” Magnetic Resonance Imaging 30, no. 9 (2012): 1323–1341, 10.1016/j.mri.2012.05.001. 22770690 PMC3466397

[34] H. Johnson , G. Harris , and K. Williams , “BRAINSFit: Mutual Information Registrations of Whole‐Brain 3D Images Using the Insight Toolkit,” Insight Journal (2007), 10.54294/hmb052.

[35] A. Roldán‐Alzate , S. García‐Rodríguez , P. V. Anagnostopoulos , S. Srinivasan , and C. J. Francois , “Kinetic Energy Efficiency of Single Ventricle and TCPC Using 4D Flow MRI,” Journal of Cardiovascular Magnetic Resonance 17, no. Suppl 1 (2015): Q97, 10.1186/1532-429X-17-S1-Q97.

[36] E. Odemis , T. Gumus , İ. B. Aka , S. Ozkok , and K. Pekkan , “Evaluation of the Total Hydrodynamic Energy Loss Using 4D Flow MRI in a Case With Fontan Failure,” Heliyon 10, no. 6 (2024): e28140, 10.1016/j.heliyon.2024.e28140. 38515711 PMC10956053

[37] T. Alsaied , L. A. Sleeper , M. Masci , et al., “Maldistribution of Pulmonary Blood Flow in Patients After the Fontan Operation Is Associated With Worse Exercise Capacity,” Journal of Cardiovascular Magnetic Resonance 20 (2018): 85, 10.1186/s12968-018-0505-4. 30558626 PMC6296022

[38] K. Jarvis , S. Schnell , A. J. Barker , et al., “Evaluation of Blood Flow Distribution Asymmetry and Vascular Geometry in Patients With Fontan Circulation Using 4‐D Flow MRI,” Pediatric Radiology 46, no. 11 (2016): 1507–1519, 10.1007/s00247-016-3654-3. 27350377 PMC5039076

[39] S. Fleming , M. Thompson , R. Stevens , et al., “Normal Ranges of Heart Rate and Respiratory Rate in Children From Birth to 18 Years of Age: A Systematic Review of Observational Studies,” Lancet 377, no. 9770 (2011): 1011–1018, 10.1016/S0140-6736(10)62226-X. 21411136 PMC3789232

[40] T. Nallamothu , E. Weiss , J. Baraboo , et al., “Respiration‐Driven Modulations in Fontan Flow Energetics: Impact of Age and General Anesthesia,” Journal of Cardiovascular Magnetic Resonance 28 (2026): 28, 10.1016/j.jocmr.2025.102316.

