# Supplementary material for: 5D Flow MRI Reveals Respiration‐Driven Changes in Blood Flow Energetics in Congenital Heart Disease
Source: Magn Reson Med. 2026 Apr 26;96(3):1342–54. doi: 10.1002/mrm.70403 (PMC13327427; doi:10.1002/mrm.70403)
Supplement: Supplementary file 1 — Table S1: Correlations with combined ventricular stroke volume in Fontan patients (*p < 0.05). Table S2: Correlations with LPA‐RPA flow differential in Fontan patients (*p < 0.05, **p < 0.01). Table S3: Correlations with left ventricular stroke volume in shunt patients (*p < 0.05). Table S4: Correlations with Qp/Qs in shunt patients (*p < 0.05). Figure S1: Impact of increased acceleration on respiratory‐driven dynamics of kinetic energy. We computed KEmean over the cardiac cycle in each respiratory state in original and accelerated data. We compared for significant differences in dynamics between paired data using repeated‐measures ANOVA followed by Tukey tests. Respiratory variability (resp var) was computed as the maximum percent change in KEmean over the respiratory cycle and compared between original and accelerated data. Significant differences are denoted (*p < 0.05, **p < 0.01, ***p < 0.001). (a) Respiratory‐driven dynamics in KEmean were significantly different with increased acceleration in the aorta of controls, and respiratory variability was decreased. KEmean dynamics were not significantly altered in (b) Fontan patients or (c) shunt patients. Figure S2: Impact of increased acceleration on respiratory‐driven dynamics of energy loss. We computed ELtotal over the cardiac cycle in each respiratory state in original and accelerated data. We compared for significant differences in dynamics between paired data using repeated‐measures ANOVA followed by Tukey tests. Respiratory variability was computed as the maximum percent change in energy loss over the respiratory cycle and compared between original and accelerated data. Significant differences are denoted (*p < 0.05, **p < 0.01, ***p < 0.001). (a) Respiratory‐driven dynamics in ELtotal were significantly different with increased acceleration in the PAs and aorta of controls, and respiratory variability was significantly decreased in both vessels. (b) ELtotal dynamics were not significantly altered in Fontan [file MRM-96-1342-s001.docx]

**Supporting Information**

Table S1: Correlations with combined ventricular stroke volume in Fontan patients (*p<0.05)


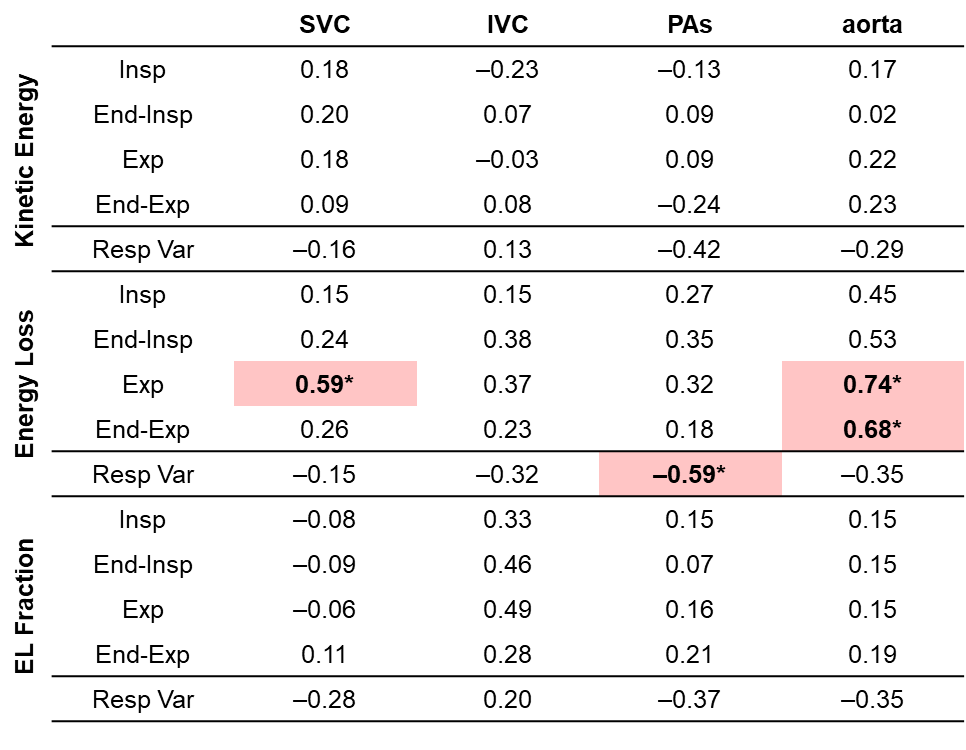


Table S2: Correlations with LPA-RPA flow differential in Fontan patients (*p<0.05, **p<0.01)


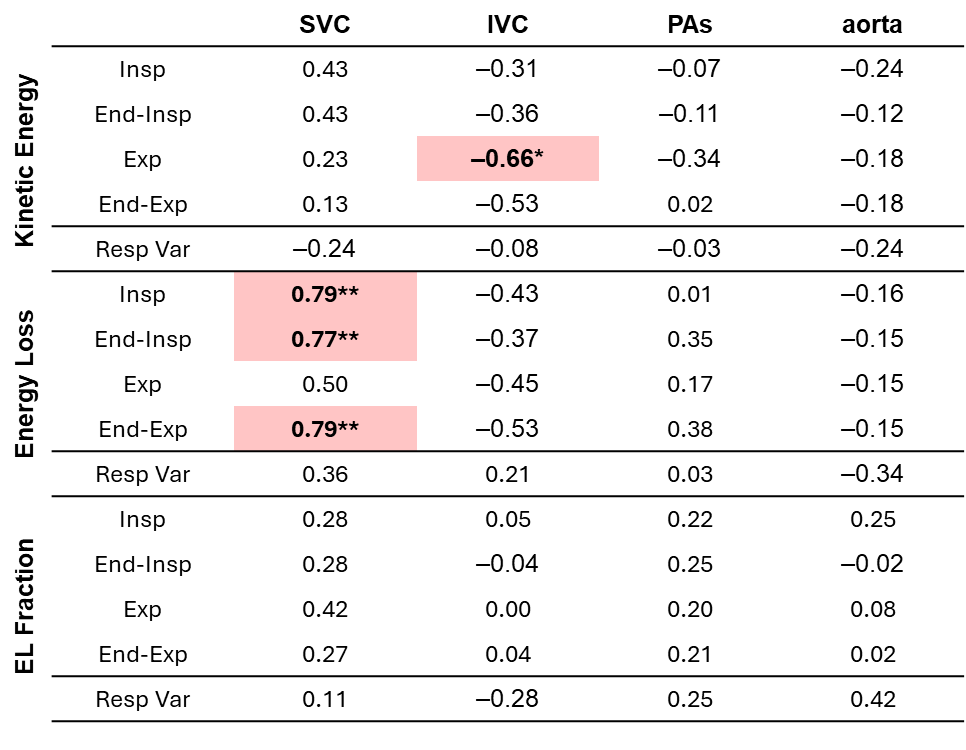


Table S3: Correlations with left ventricular stroke volume in shunt patients (*p<0.05)


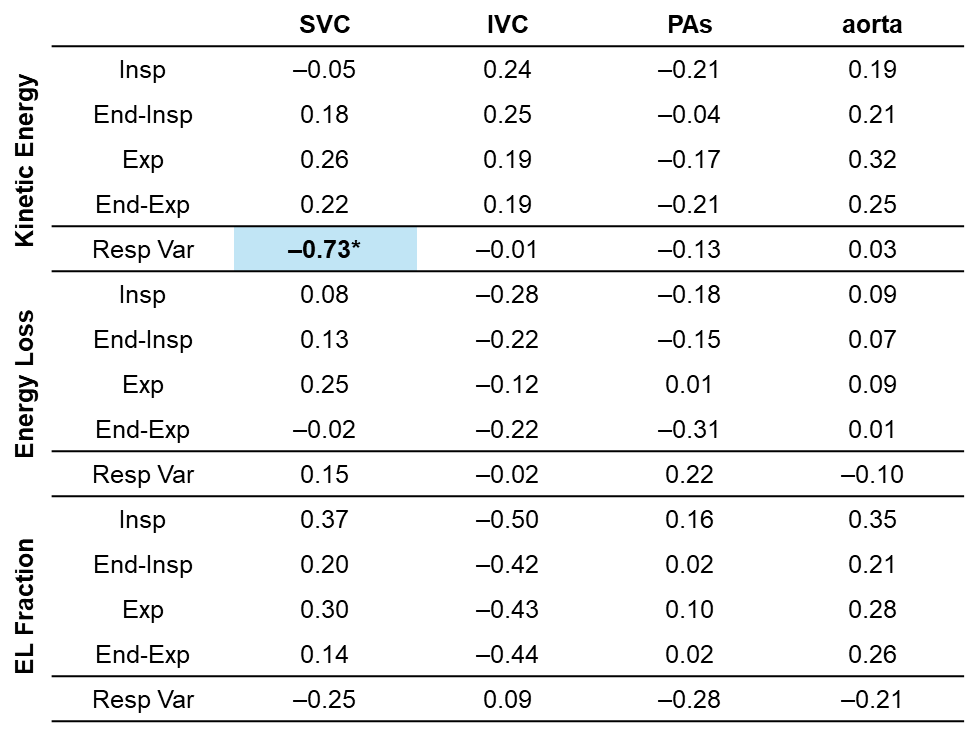


Table S4: Correlations with Qp/Qs in shunt patients (*p<0.05)


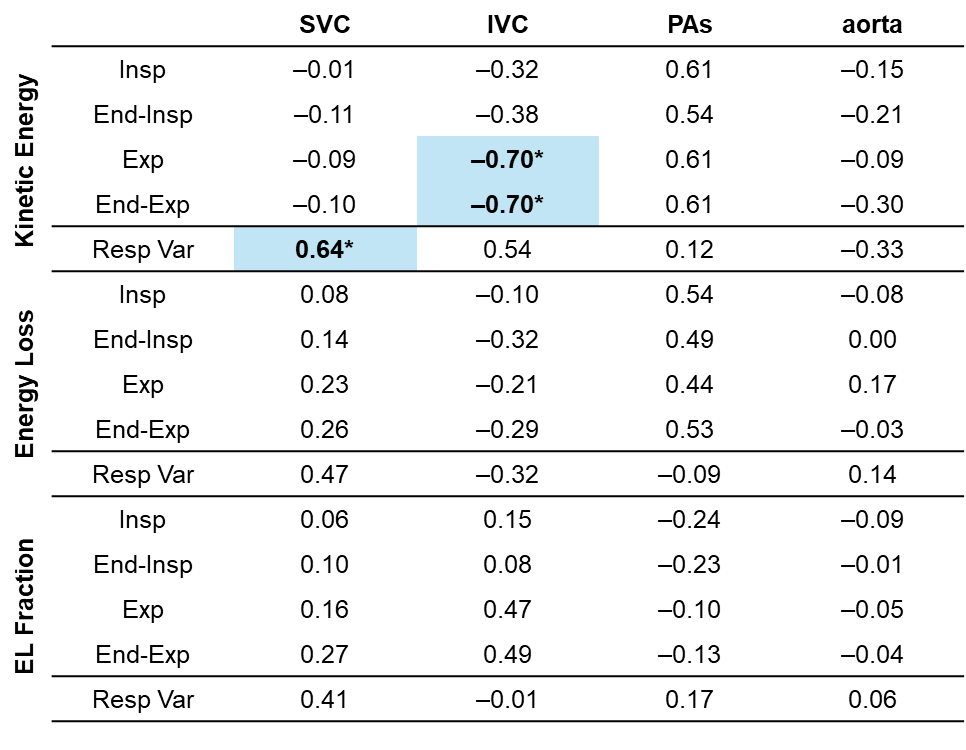


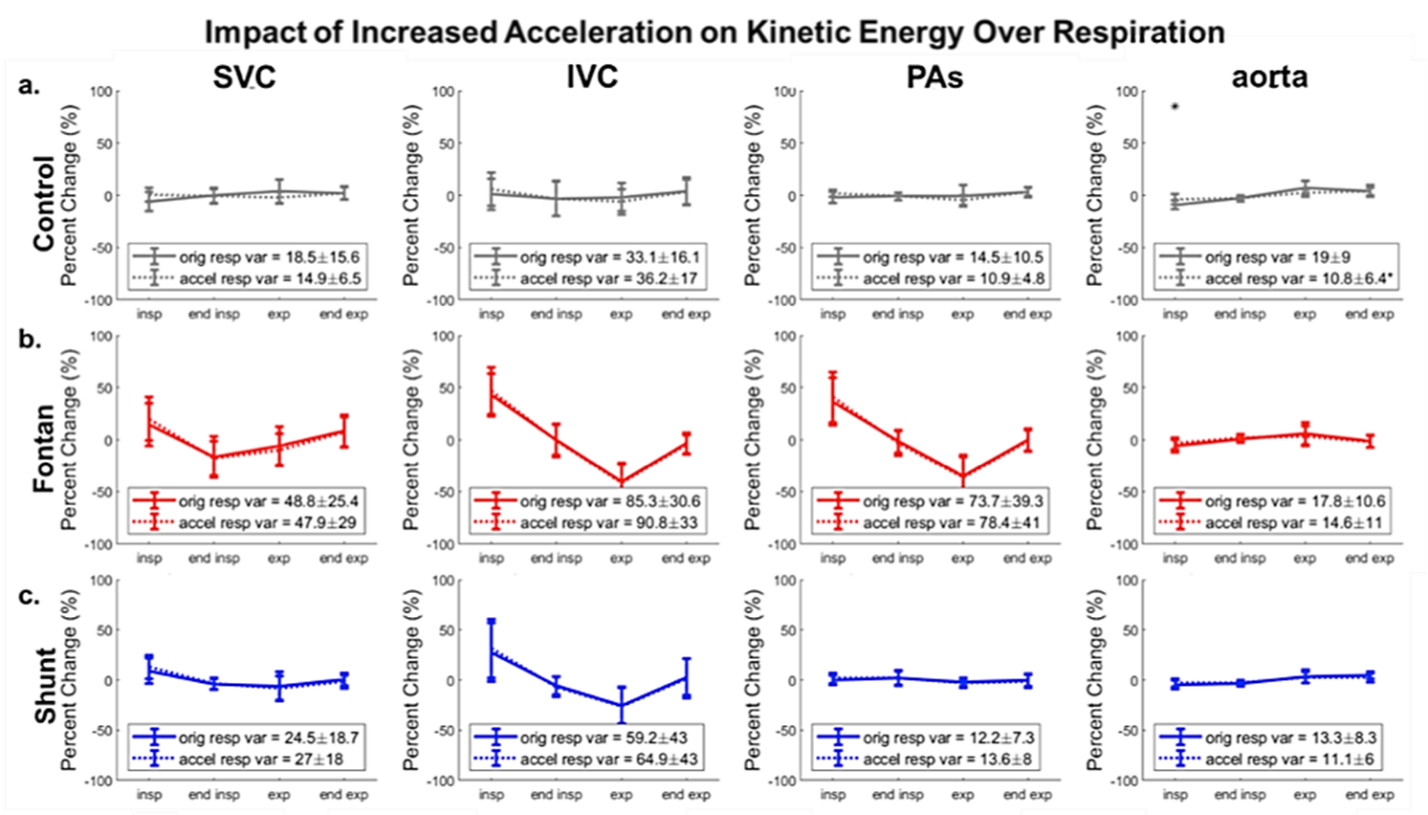
 Figure S1: Impact of increased acceleration on respiratory-driven dynamics of kinetic energy. We computed KE_mean_ over the cardiac cycle in each respiratory state in original and accelerated data. We compared for significant differences in dynamics between paired data using repeated-measures ANOVA followed by Tukey tests. Respiratory variability (resp var) was computed as the maximum percent change in KE_mean_ over the respiratory cycle and compared between original and accelerated data. Significant differences are denoted (*=p<0.05, **=p<0.01, ***=p<0.001). (a) Respiratory-driven dynamics in KE_mean_ were significantly different with increased acceleration in the aorta of controls, and respiratory variability was decreased. KE_mean_ dynamics were not significantly altered in (b) Fontan patients or (c) shunt patients.


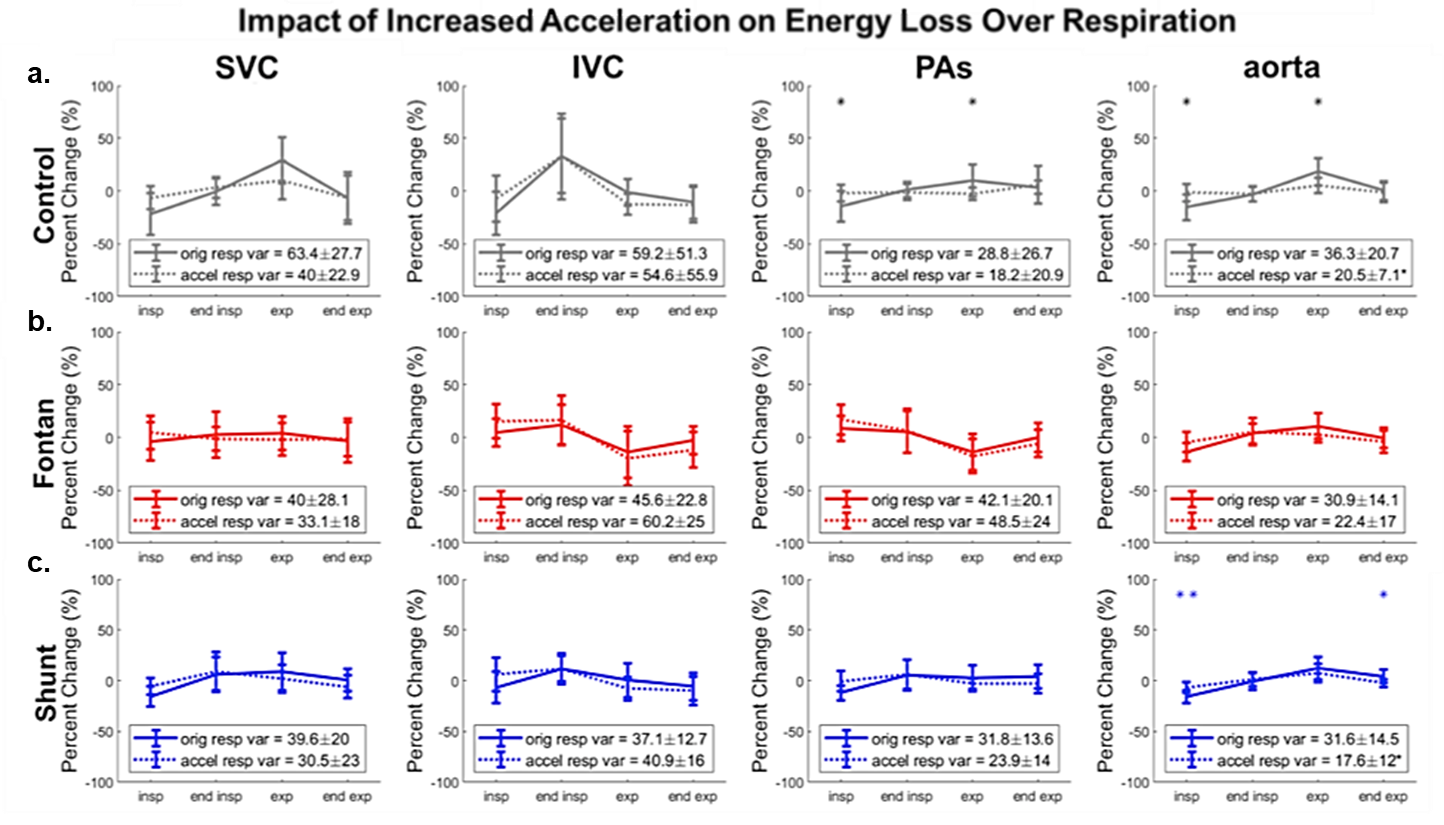


Figure S2: Impact of increased acceleration on respiratory-driven dynamics of energy loss. We computed EL_total_ over the cardiac cycle in each respiratory state in original and accelerated data. We compared for significant differences in dynamics between paired data using repeated-measures ANOVA followed by Tukey tests. Respiratory variability was computed as the maximum percent change in energy loss over the respiratory cycle and compared between original and accelerated data. Significant differences are denoted (*=p<0.05, **=p<0.01, ***=p<0.001). (a) Respiratory-driven dynamics in EL_total_ were significantly different with increased acceleration in the PAs and aorta of controls, and respiratory variability was significantly decreased in both vessels. (b) EL_total_ dynamics were not significantly altered in Fontan patients. (c) Respiratory variability was decreased in the aorta of shunt patients.


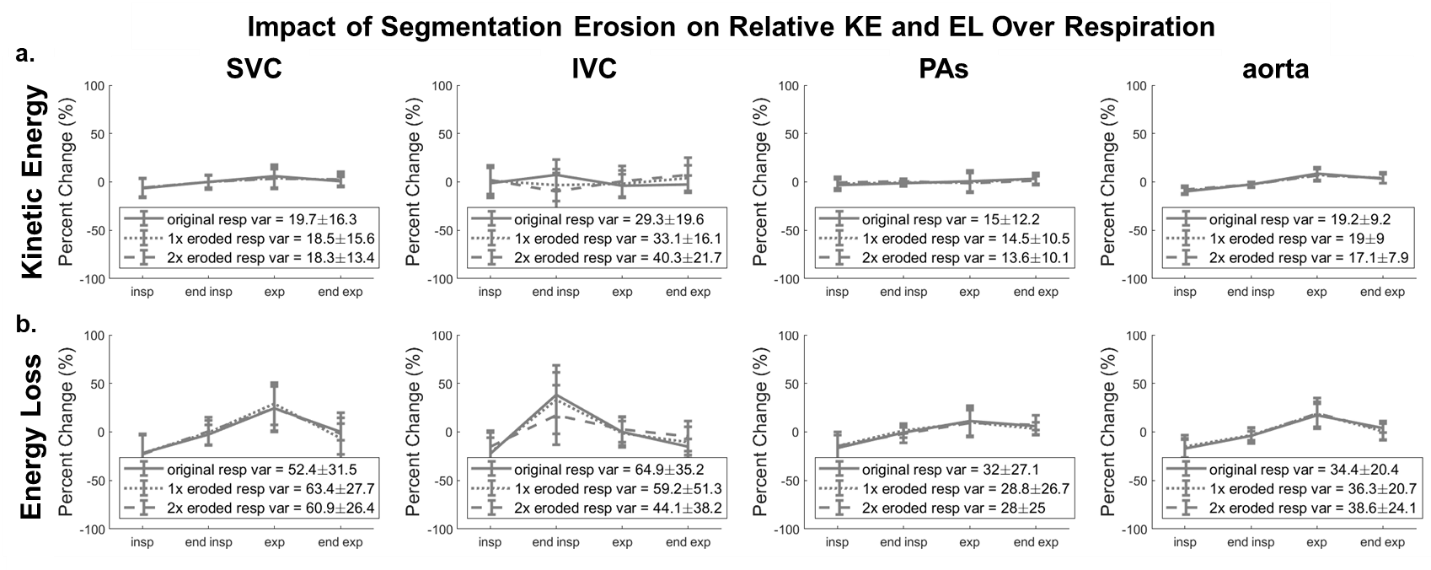


Figure S3: Impact of segmentation erosion on respiratory-driven dynamics of kinetic energy and energy loss. In controls, we computed relative changes in KE_mean_ and EL_total_ over the cardiac cycle in each respiratory state in original segmentations and segmentations eroded by spherical elements with radius 1 voxel (1x) and 2 voxels (2x). We compared for significant differences in dynamics between paired data using repeated-measures ANOVA followed by Tukey tests. Respiratory variability (resp var) was computed as the maximum percent change in each metric over the respiratory cycle and compared between original and accelerated data. No significant differences were found in relative (a) KE_mean_ or (b) EL_total_.


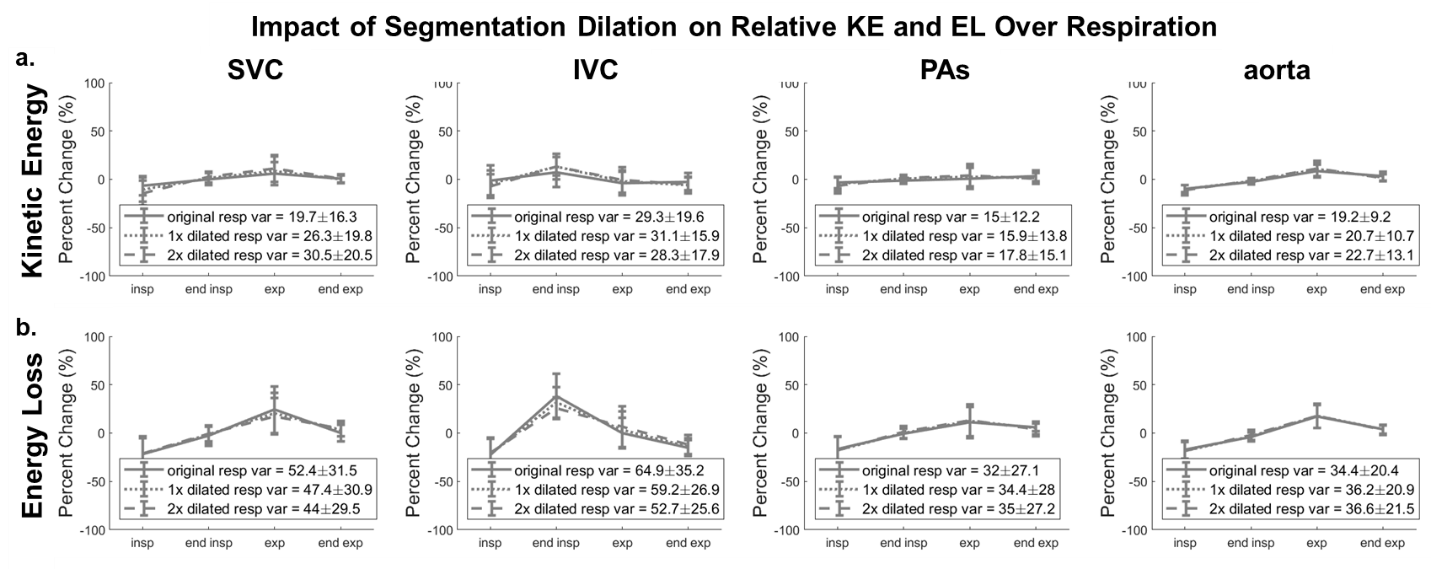


Figure S4: Impact of segmentation dilation on respiratory-driven dynamics of kinetic energy and energy loss. In controls, we computed relative changes in KE_mean_ and EL_total_ over the cardiac cycle in each respiratory state in original segmentations and segmentations dilated by spherical elements with radius 1 voxel (1x) and 2 voxels (2x). We compared for significant differences in dynamics between paired data using repeated-measures ANOVA followed by Tukey tests. Respiratory variability (resp var) was computed as the maximum percent change in each metric over the respiratory cycle and compared between original and accelerated data. No significant differences were found in relative (a) KE_mean_ or (b) EL_total_.

**Sensitivity to Segmentation Vessel Boundary:**

It is possible that including segmentation registration in the image analysis workflow could introduce spurious inclusion/exclusion of voxels at the segmentation boundaries that vary over respiratory states. Viscous energy loss is computed with velocity derivatives, making this metric particularly sensitive to noise at boundary voxels. To investigate whether our measurements of relative changes in KE_mean_ or EL_total_ over the respiratory cycle are influenced by inclusion/exclusion of boundary voxels, we computed these metrics with segmentations eroded and dilated by 1-2 voxels and compared for significant differences in relative dynamics in each respiratory stat.

Our results indicated that neither erosion (Figure S3) nor dilation (Figure S4) significantly impacted the dynamics of relative KE_mean_ or EL_total_ over the respiratory cycle. This indicates that our measurements were robust to registration-induced segmentation boundary differences. Moderate (but not statistically significant) differences were seen in the IVC with an erosion by two voxels, likely due to the small size of this segmentation and reduction to a volume of only a few voxels.
